# Predictive factors of occupational stress among nurses during the COVID-19 pandemic: a cross-sectional study in Kashan, Iran

**DOI:** 10.1186/s12912-024-01967-0

**Published:** 2024-05-09

**Authors:** Zahra Ghaderi, Zahra Tagharrobi, Zahra Sooki, Khadijeh Sharifi

**Affiliations:** 1 Department of Nursing, Trauma Nursing Research Center, Faculty of Nursing and Midwifery, Kashan University of Medical Sciences, Kashan, Iran; 2 Faculty of Nursing and Midwifery, Trauma Nursing Research Centre, Kashan University of Medical Sciences, Kashan, IR Iran

**Keywords:** COVID-19, Iranian nurses, Occupational stress

## Abstract

**Background:**

Considering the severe and sudden changes in the job conditions of nurses during the covid-19 pandemic, the increase in job tensions during this critical period and its consequences on the quantity and quality of nursing care, this study aims to investigate the job stress of nurses during the covid-19 pandemic and its predictors in Iran.

**Methods:**

This cross-sectional study was conducted on 400 nurses in ‘Kashan’, Iran, who were randomly selected using stratified sampling. Data were collected using two questionnaires on occupational stress and potential related factors. The data were analyzed in SPSS version 16.

**Results:**

The results showed that the occupational stress of nurses in Iran was at a medium to high level with a mean and standard deviation of 103.773 ± 15.742 (scale of 34–136). Factors such as satisfaction with physical health, quality of work life, satisfaction with the availability of facilities, sense of coherence, education level, work experience, job burnout, male gender, being native, and workplace were predictors of occupational stress and explained 23.3% of the variance in occupational stress score. The highest contribution was related to satisfaction with physical health.

**Conclusion:**

Considering the consequences of occupational stress for nurses, it is crucial for health and nursing authorities to take these factors into consideration in policy-making and planning.

**Supplementary Information:**

The online version contains supplementary material available at 10.1186/s12912-024-01967-0.

## Background

Occupational stress is an emotional, cognitive, behavioral, and psychological response to the harmful aspects of the work environment [1]. It occurs when the expectations from an individual exceed their abilities and capabilities [2]. Nurses, due to the nature of their profession, are constantly exposed to various occupational stressors. The level of occupational stress was reported to be 124.06 ± 32.58 (scale of 46–201) in Chaudhari et al.‘s (2018) study in India [3], 66.2% in Baye et al.‘s (2020) study in Ethiopia [4], and 109.06 ± 16.22 (scale of 175 − 35) in Ghadirzadeh and Adib-Hajbaghery’s (2017) study in Iran [5].

With the onset of the COVID-19 pandemic, nurses experienced unprecedented levels of occupational stress due to being at the forefront of the fight against this unknown disease with high mortality rates. Among these stresses were shortages of medical facilities and equipment [6], wearing heavy protective clothing and masks for long periods [7], extremely high work pressure, long working hours, cancellation of many personal and recreational programs, being away from family, limited close contact with family and friends, and concern about transmitting the disease to loved ones [2, 8]. Some studies reported nurses’ occupational stress during the COVID-19 pandemic in different amount; in Saudi Arabia 6.92 ± 2.91 (scale of 0–10) [9], in Jordan 94.59 ± 6.08 (scale of 34–136) [10], in China 91.42 ± 26.09 (scale of 35–140) [2], in Egypt 193.55 ± 44.94 (scale of 57–228) [11], and in Iran 72% [12].

During the COVID-19 pandemic, nurses experienced significant job-related stress, which resulted in various negative consequences such as reduced efficiency, decreased attention and focus, impaired decision-making skills, increased absenteeism, decreased organizational commitment, reduced job satisfaction [13], an increased tendency to quit [14] and burnout [15], a higher likelihood of clinical errors and decreased quality of nursing care [4].

Studies on factors related to occupational stress indicate various contradictory factors. Some studies have shown a significant statistical relationship between occupational stress and marital status [16], gender, workplace, work experience [17, 18], work overload [19], shift work, mental health [17], education level [5, 20], number of children [21], role ambiguity, job change, job satisfaction, spiritual health [22], quit job, quality of work life [14], job burnout, resilience [23], and sense of coherence [24].

Some studies have not shown a significant statistical relationship between occupational stress and age, marital status [17, 21], coworker support [19], education level, patient safety [16], gender, work experience [21], work shifts, job position, income [5], workplace, overtime hours, and number of children [20].

Considering the consequences of nurses’ occupational stress and the impact of the environment and structure on the level of stress and changes in working conditions during pandemics such as COVID-19, and the contradictions in previous studies regarding related factors, this study was conducted.

## Methods

### Study design

This cross-sectional study was conducted between August and September 2022 (Simultaneously with one of the peak of COVID-19 in ‘Kashan’, Iran) in hospitals affiliated to ‘Kashan’ University of Medical Sciences in Iran.

### Sampling method

The study population included all nurses who were employed in seven hospitals affiliated to ‘Kashan’ University of Medical Sciences in Iran. To determine the sample size, a previous study by Farhadi et al. (2013) was consulted, which reported occupational stress using the Taft and Anderson questionnaire as 121.36 ± 19.88 [25]. Based on formula number one and considering the accuracy of one tenth of a standard deviation, the sample size was estimated to be 383 individuals. However, considering potential attrition, 400 individuals were selected for the study using stratified random sampling based on hospital wards in all seven hospitals.

Formula number 1

$$\frac{{\mathbf{S}}^{2}{\mathbf{Z}}^{2}}{{\mathbf{d}}^{2}}$$

### Inclusion and exclusion criteria

The inclusion criteria for participants were being employed in clinical activities in hospital wards (inpatient departments), having at least one year of work experience in clinical settings, willingness to participate in the study, not having any known psychological disorders (self-reported), and having a university degree in nursing. The exclusion criteria were dropping out of the study during questionnaire completion and providing incomplete responses to the questionnaires.

### Measures

The data collection tools consisted of two questionnaires: The Personal and Occupational Characteristics Questionnaire and “the Gary Toft and Anderson’s Job stress Questionnaire” Especially for nurses.

The first questionnaire, a 36-item questionnaire on personal and occupational characteristics, was developed by the researcher based on a review of the literature. The qualitative content validity of this questionnaire was confirmed by six faculty members from the Nursing and Midwifery department at ‘Kashan’ University of Medical Sciences in Iran. The questionnaire was consisted of two sections: personal characteristics (17 questions) and occupational characteristics (19 questions). Personal characteristics included gender, marital status, age, education level, family income, number of children, ethnicity, regular exercise, sleep and nutrition status, religious beliefs and adherence, history of COVID-19 infection, history of COVID-19 infection in family members, family and friend support and satisfaction with physical and mental health. The occupational characteristics section included workplace, clinical work experience, experience working in COVID-19 wards and temporary COVID-19 wards (inpatient and temporary hospitalization departments for COVID-19 patients), amount of overtime work per month, dominant work shift, quality of work life, interest in nursing profession, job satisfaction, satisfaction with staffing levels in each shift, job burnout, work-family conflict, satisfaction with salary and wages, physician’s behavior and performance towards oneself, colleagues’ behavior and performance towards oneself, satisfaction with behavior and performance of head nurse towards oneself, nursing office officials’ behavior and performance towards oneself, patient feedback, and satisfaction with availability of medical equipment and facilities.

The second questionnaire was the Gary Taft and Anderson Nursing Stress Scale, which consisted of 34 questions in 7 domains [26]. Responses to the items were rated on a Likert scale as not-stressful (1 point), rarely stressful (2 points), sometimes-stressful (3 points), and always-stressful (4 points). The total score ranged from 34 to 136, with higher scores indicating greater levels of stress. The reliability and validity of this questionnaire have been established in the Iranian population, with a Cronbach’s alpha coefficient of 0.77 reported by Salemi et al. (2017) [27]. In the current study, both Cronbach’s alpha and McDonald’s omega were calculated to be 0.934.

### Data collection method

The first author visited the nursing offices of each of the seven hospital and obtained permission to conduct the research. A list of all departments where nurses were clinically active, along with the number and names of nurses employed in those departments, was obtained. Nurses were randomly selected from each department based on the inclusion criteria and desired sample size. The researcher visited each department at the beginning of each shift, explained the research, and obtained written and verbal consent from eligible nurses. Necessary explanations on how to complete the questionnaires were provided, and the questionnaires were collected at the end of the shift. In cases where the questionnaire was not completed on the scheduled date, an agreement was made with the nurse regarding the delivery time. Each nurse was assigned a unique code.

### Data analysis

Data analysis was performed using SPSS software version 16. Measures of central tendency and dispersion were used to describe quantitative variables, while absolute and relative frequencies were used for categorical variables. Skewness and kurtosis indices were used to test for normality of quantitative data, with a range of ± 2 considered as normal.

The collected data were analyzed in two stages. In the first stage, univariate tests were used to examine the relationship between each potential categorical factor. Independent t-tests were used for dichotomous factors, and one-way analysis of variance (ANOVA) or non-parametric equivalent (Kruskal-Wallis) was used for non-normal data. Pearson correlation coefficient was used for quantitative factors. In the second stage, multiple stepwise linear regression analysis was conducted to investigate the precise role of potential factors in determining variations in the occupational stress score, while removing the confounding effect of other factors. Variables that were significant in the univariate analysis (with a P-value < 0.2) were included in the multiple regression analysis. A significance level of less than 0.05 was considered in all analyses.

Missing data for the item “satisfaction with the behavior and performance of the head nurse” were replaced with the mean score. Additionally, missing data for the item “satisfaction with the performance of the head nurse” (for nurses who had served as head nurses for a long time) were also replaced with the mean score.

## Results

The questionnaires were distributed among 400 nurses working in the hospitals affiliated to ‘Kashan’ University of Medical Sciences, and all the questionnaires were analyzed. Overall, 76% of the participants were female, 83.3% were married, and 84.8% had a bachelor’s degree. The mean age of the participants was 34.41 ± 7.17 years, and the mean work experience was 10.29 ± 6.86 years. The mean level of interest in nursing profession, satisfaction with the behavior of nursing office officials, and satisfaction with salary and benefits were approximately 7.43, 5.85, and 3.61 (scale of 0–10), respectively. The mean score of nursing stress among the participants was 103.773 ± 15.742 (scale of 34–136). The results of the Univariate analysis showed that there was a statistically significant relationship between age, gender, education, employment status, being native, number of children, regular exercise, nutritional status, workplace, job position, work experience in the COVID-19 ward, work experience in the temporary COVID-19 ward, work-family conflict, support from friends, satisfaction with the number of personnel in each shift, job satisfaction, quality of work life, job burnout, satisfaction with physical and mental health, satisfaction with the behavior of physicians, satisfaction with the behavior of colleagues, satisfaction with the behavior of nursing office officials, satisfaction with feedback from patients, satisfaction with the availability of facilities and equipment, and sense of coherence with nursing stress (Tables 1 and 2).

The multiple linear regression model showed that the simultaneous presence of 10 variables in the model was significant in predicting nursing occupational stress. Satisfaction with physical health, quality of work life, satisfaction with facilities and equipment, and sense of coherence had a negative effect on occupational stress. Education level, work experience, and job burnout had a positive effect on nursing occupational stress. Being male and native were associated with lower levels of occupational stress. Workplace was also a significant factor affecting occupational stress (P-value < 0.001, F = 11.848). Additionally, this analysis showed that 23.3% of the variance in nursing occupational stress scores was explained by these 10 variables, with the greatest contribution coming from satisfaction with physical health (R2 = 0.078) (Table 3).

**Table 1:** Occupational stress scores of nurses categorized by personal and job variables

Variables		Mean ± SD	Test type	*P*-value
Gender	Male	99.010 ± 16.274	t = 3.446^a^	0.001
	Female	105.276 ± 15.292		
Marital status	Single	102.323 ± 17.242	f = 0.379^b^	0.685
	Married	104.075 ± 15.460		
	Divorced	100.500 ± 17.678		
Education level	Associate degree	90.000 ± 21.342	test statistic = 9.311^c^	0.010
	Bachler’s degree	103.056 ± 15.917		
	Master’s degree	109.339 ± 12.477		
Being native	No	109.353 ± 17.668	t = − 2.171^a^	0.031
	Yes	103.254 ± 15.476		
Regular exercise	No	104.986 ± 15.029	t = − 2.241^a^	0.026
	Yes	100.975 ± 17.012		
Sleep status	Completely inappropriate (0)	106.048 ± 16.612	f = 1.678^b^	0.154
	Slightly appropriate (1)	103.032 ± 17.537		
	Relatively appropriate (2)	104.349 ± 14.478		
	Very appropriate (3)	97.393 ± 16.324		
	Completely appropriate (4)	99.00 ± 5.292		
Nutrition status	Completely inappropriate (0)	105.846 ± 18.010	f = 4.409^b^	0.002
	Slightly appropriate (1)	107.208 ± 14.065		
	Relatively appropriate (2)	103.768 ± 15.211		
	Very appropriate (3)	96.500 ± 18.197		
	Completely appropriate (4)	93.333 ± 14.052		
Adherence to beliefs	Not at all (0)	104.143 ± 16.896	test statistic = 0.201^c^	0.936
	Weak (1)	100.063 ± 19.317		
	Average (2)	103.578 ± 14.200		
	High (3)	104.538 ± 18.103		
	Very high (4)	103.975 ± 15.051		
Workplace	Emergency	102.164 ± 15.398	test statistic = 32.210^c^	0.009
	Internal	104.193 ± 14.206		
	Surgical	100.867 ± 17.368		
	Pediatric	100.417 ± 18.812		
	ICU	104.228 ± 16.132		
	CCU	105.350 ± 13.484		
	Dialysis	99.375 ± 17.629		
	Obstetrics and Gynecology	102.500 ± 16.521		
	Operating Room	106.800 ± 15.796		
	Neonatology	107.100 ± 7.564		
	NICU	121.400 ± 8.809		
	Angiography	101.375 ± 16.265		
	Psychiatric Emergency	97.143 ± 16.028		
	Adult Psychiatric Inpatient	111.375 ± 12.961		
	Child Psychiatric Inpatient	98.833 ± 13.920		
	COVID	104.400 ± 15.792		
	Ophthalmology and ENT	115.00 ± 10.559		
Experience working in COVID-19 inpatient wards	No	107.908 ± 13.266	t = − 2.327^a^	0.020
	Yes	102.970 ± 16.073		
Experience working in COVID-19 temporary wards	No	107.853 ± 13.253	t = − 2.507^a^	0.013
	Yes	102.831 ± 16.134		
History of COVID-19 infection	No	107.111 ± 14.136	t = − 1.680^a^	0.094
	Yes	103.251 ± 15.933		
History of COVID-19 infection in family members	No	105.133 ± 15.353	t = − 0.492^a^	0.623
	Yes	103.662 ± 15.788		
Dominant work shift	Morning	104.870 ± 16.325	f = 2.900^b^	0.056
	Evening	105.738 ± 14.086		
	Night	101.439 ± 16.098		
Work-family conflict	No	100.047 ± 16.968	t = 4.067^a^	< 0.0001
	Yes	106.555 ± 14.171		
Family income	Insufficient	103.296 ± 17.642	f = 0.127^b^	0.883
	Sufficient	104.008 ± 14.662		
	More than sufficient	106.333 ± 12.662		
Family support	None	106.867 ± 19.310	f = 2.020^b^	0.091
	Low	106.333 ± 15.228		
	Moderate	105.446 ± 14.876		
	High	101.771 ± 15.573		
	Very high	100.828 ± 15.976		
Friend support	None	107.327 ± 15.293	f = 4.137^b^	0.003
	Low	105.939 ± 16.772		
	Moderate	102.447 ± 16.772		
	High	104.381 ± 14.186		
	Very high	93.091 ± 20.789		

^a^Independent t-test ^b^ANOVA ^c^Kruskal–Wallis

**Table 2:** Occupational stress score of nurses categorized by quantitative personal and occupational variables and sense of coherence

Variables	Correlation coefficient^a^	Result
Age (years)	0.168	0.001
Number of children	0.108	0.031
Work experience (years)	0.184	< 0.0001
Overtime per month (hours)	− 0.077	0.122
Satisfaction with the adequacy of the number of personnel in each shift (scale of 0–10)	-0.108	0.030
Interest in the nursing profession (scale of 0–10)	0.014	0.786
Job satisfaction (scale of 0–10)	-0.149	0.003
Quality of work life (scale of 0–10)	-0.198	< 0.0001
Job burnout (scale of 0–10)	0.134	0.007
Satisfaction with salary and wages (scale of 0–10)	-0.052	0.296
Satisfaction with physical health status (scale of 0–10)	-0.280	< 0.0001
Satisfaction with mental health status (scale of 0–10)	-0.279	< 0.0001
satisfaction with the behavior and performance of physicians towards oneself (scale of 0–10)	-0.191	< 0.0001
satisfaction with the behavior and performance of colleagues towards oneself (scale of 0–10)	-0.182	< 0.0001
satisfaction with the behavior and performance of head nurse towards oneself (*n* = 390) (scale of 0–10)	-0.056	0.270
satisfaction with the behavior and performance of nursing office officials towards oneself (scale of 0–10)	-0.152	0.002
satisfaction with patient feedback (scale of 0–10)	-0.123	0.014
Satisfaction with availability of medical equipment and facilities (scale of 0–10)	-0.171	0.001
the sense of coherence	-0.170	0.001

^a^Pearson

**Table 3:** Results of multiple linear regression on factors affecting occupational stress score

Model	B	95% CI B		SE	Beta	t value	*p*-value
		lower bound	Upper bound				
constant	120.076	105.210	134.942	7.561	-	15.881	< 0.0001
Satisfaction with physical health	-0.864	-1.538	-0.190	0.343	-0.131	-2.521	0.012
education	4.969	1.139	8.789	1.948	0.117	2.551	0.011
Gender	-6.313	-9.614	-3.012	1.679	-0.171	-3.760	< 0.0001
Work experience	0.519	0.304	0.734	0.110	0.226	4.740	< 0.0001
Quality of work life	-0.856	-1.572	-0.141	0.364	-0.125	-2.354	0.019
Being native	-8.291	-13.283	-3.300	2.539	-0.147	-3.266	0.001
Job burnout	0.724	0.139	1.309	0.298	0.109	2.435	0.015
Satisfaction with availability of medical equipment and facilities (scale of 0–10)	-0.892	-1.573	-0.210	0.347	-0.130	-2.573	0.010
Workplace	0.361	0.054	0.667	0.156	0.108	2.314	0.021
the sense of coherence	-1.680	-0.313	-0.023	0.074	-0.106	-3.281	0.023

*Note* R^2^ = 0.078 Adjusted R^2^ = 0/233 F = 11/848 *P* < 0/0001

*The missing items of ten questionnaires related to “Satisfaction with the Performance of the Head Nurse” (which was answered by nurses who had been in the position of head nurse for a long time) was replaced with a mean

## Discussion

The present study aimed to investigate the predictors of nursing occupational stress. The findings showed that the mean total score of nursing occupational stress was 103.773 ± 15.742 (scale of 34 to 136), indicating a higher-than-average level of occupational stress among nurses during the COVID-19 pandemic. The results of the study by Zhan et al. (2020) were consistent with the present study, showing moderate to high levels of occupational stress among Chinese nurses during the pandemic [2]. Tayyib and Alsulami (2020) also reported higher than average levels of occupational stress among nurses in Saudi Arabia during this period [9]. The findings of the study by Alkhawaldeh et al. (2020) in Jordan showed moderate levels of occupational stress among nurses during this outbreak [10]. However, Said and El-Shafei (2021) reported high levels of nursing occupational stress in Egypt [11]. Nursing occupational stress has been consistently found to be moderate to higher than average and in fact, it has been worthy of attention in all studies during the COVID-19 pandemic. Differences may be influenced by variations in shift work and workload, societal expectations, workplace stressors, amount of rest and free time, time spent with family, friends, and engaging in group and physical activities [28–30], as well as the number of pandemic-related deaths in the region and availability of medical and protective equipment [6].

The results also show that nursing occupational stress has a significant relationship with gender, education level, employment status, native status, regular exercise, nutrition status, workplace, job position, experience in COVID-19 ward, experience in temporary COVID-19 hospitalization ward, work-family conflict and social support, age, number of children, satisfaction with staffing levels in each shift, work satisfaction, quality of work life, job burnout, satisfaction with physical and mental health, behavior of physicians, behavior of colleagues, the behavior of nursing office officials, satisfaction with patient feedback, availability of facilities and equipment, and sense of coherence.

The results of the multivariate analysis showed that satisfaction with physical health, quality of work life, availability of facilities, sense of coherence, education level, work experience, job burnout, male gender, native status, and workplace were influential factors on nursing occupational stress. Additionally, the multivariate analysis indicated that 23.3% of the variance in nursing occupational stress scores could be explained by the 10 aforementioned variables, with the greatest contribution coming from satisfaction with physical health. Consistent with the findings of this study, Hendy et al. (2021) reported that during the pandemic, factors predicting nursing occupational stress in Egypt included workplace, education level, participation in COVID-19-related training courses, fear of infection, fear of transmitting the disease to family members, unavailability of protective equipment, performance of officials, nurse shortage, and the stigma of COVID-19 [31].

The results of the study by Zhan et al. (2020) in China also showed a significant correlation between nursing occupational stress levels and daily working hours, number of night shifts per week, work experience, and education level [2]. Jamali et al. (2012) in Mashhad, Iran also reported that nursing occupational stress was related to gender, education level, and the level of nurse’s awareness [32].

The most influential factor on the level of nursing occupational stress in this study was satisfaction with physical health status. Several studies in this field have indicated that physical health is a significant source of stress for nurses. Moreover, working in an environment that does not prioritize their well-being can have a detrimental effect on their physical health, as evidenced by research conducted prior to the COVID-19 outbreak. Unfortunately, the pandemic has only served to exacerbate and intensify this pre-existing issue [32, 33]. The findings of the Melnyk study (2022) indicated that during the pandemic, only 25% of nurses had good physical health status, and more than 50% of them reported that COVID-19 pandemic impacted their physical health negatively [34], which can lead to increased occupational stress, absenteeism, and a decrease in the quantity and quality of nursing care.

### Study strengths and limitations

The present study has several strengths, including a relatively large sample size, stratified sampling, and the use of multivariate analysis alongside univariate analysis. However, there are limitations to consider. For instance, some variables, such as work-family conflict, were measured using only one question.

### Conclusions and implication

In the multiple linear regression model, 10 variables including satisfaction with physical health, work-life quality, satisfaction with facilities, sense of coherence, education level, work experience, job burnout, gender (male), being native, and workplace explained 23.3% of the variance in nurses’ occupational stress score, with the greatest contribution coming from satisfaction with physical health. Given that healthcare systems worldwide faced challenges during the COVID-19 pandemic, and there were sudden and significant changes in nurses’ work conditions, and also in order to prepare for crises and problems that threaten the life of humanity it is essential for researchers to investigate occupational stress and related factors among nurses during the pandemic to enable health system officials and planners to implement appropriate interventions to improve working conditions.

### Electronic supplementary material

Below is the link to the electronic supplementary material.

Supplementary Material 1

## Data Availability

The datasets used and/or analyzed during the current study are available from the corresponding author upon reasonable request.
