# Supplementary material for: Predictive factors of occupational stress among nurses during the COVID-19 pandemic: a cross-sectional study in Kashan, Iran
Source: BMC Nurs. 2024 May 9;23:313. doi: 10.1186/s12912-024-01967-0 (PMC11083832; doi:10.1186/s12912-024-01967-0)
Supplement: Supplementary file 1 — Supplementary Material 1 [file 12912_2024_1967_MOESM1_ESM.docx]

**Questionnaire of factors related to occupational stress of nurses**

**Dear nurse,**

**This questionnaire was organized in connection with a research aimed at “determining the occupational stress status of nurses during the covid pandemic”. Please read the questions carefully and write or mark your answer with a cross.**

**Researcher: Dr. Khadijeh. Sharifi, faculty member of nursing and midwifery faculty, Kashan University of Medical Sciences**

| 1 | Age (in year) | … |
| --- | --- | --- |
| 2 | gender | Female□ male□ |
| 3 | Marital status | Single□ Married□ Divorced□ |
| 4 | Number of children | … |
| 5 | Education level | Associate degree□ Bachler's degree□ Master's degree□ |
| 6 | Being native | No□ Yes□ |
| 7 | Regular exercise (At least 3 times a week) | No□ Yes□ |
| 8 | Sleep status | Completely inappropriate □  Slightly appropriate□  Relatively appropriate □  Very appropriate □  Completely appropriate □ |
| 9 | Nutrition status | Completely inappropriate □  Slightly appropriate□  Relatively appropriate□  Very appropriate□  Completely appropriate□ |
| 10 | Adherence to beliefs | Not at all□ Weak□ Average□ High□ Very high □ |
| 11 | Workplace | Emergency□  Internal□  Surgical□  Pediatric□  ICU□  CCU□  Dialysis□  Obstetrics and Gynecology□  Operating Room□  Neonatology□  NICU□  Angiography□  Psychiatric Emergency□  Adult Psychiatric Inpatient□  Child Psychiatric Inpatient□  COVID-19□  Ophthalmology and ENT□ |
| 12 | Work experience (in years) | … |
| 13 | Experience working in COVID-19 inpatient wards | No□ Yes□ |
| 14 | Experience working in COVID-19 temporary wards | No□ Yes□ |
| 15 | History of COVID-19 infection | No□ Yes□ |
| 16 | History of COVID-19 infection in family members | No□ Yes□ |
| 17 | Dominant work shift | Morning□ Evening□ Night□ |
| 18 | Satisfaction with the adequacy of the number of personnel in each shift (scale of 0-10) | … |
| 19 | Interest in the nursing profession (scale of 0-10) | … |
| 20 | Job satisfaction (scale of 0-10) | … |
| 21 | Quality of work life (scale of 0-10) | … |
| 22 | Job burnout (scale of 0-10) | … |
| 23 | Work-family conflict | No□ Yes□ |
| 24 | Overtime per month (in hours) | … |
| 25 | Family income | Insufficient□ Sufficient□ More than sufficient□ |
| 26 | Satisfaction with salary and wages (scale of 0-10) |  |
| 27 | Family support | None□ Low□ Moderate□ High□ Very high □ |
| 28 | Friend support | None□ Low□ Moderate□ High□ Very high □ |
| 29 | Satisfaction with physical health status (scale of 0-10) | … |
| 30 | Satisfaction with mental health status (scale of 0-10) | … |
| 31 | satisfaction with the behavior and performance of physicians towards oneself (scale of 0-10) | … |
| 23 | satisfaction with the behavior and performance of colleagues towards oneself (scale of 0-10) | … |
| 33 | satisfaction with the behavior and performance of head nurse towards oneself (n=390) (scale of 0-10) | … |
| 34 | satisfaction with the behavior and performance of nursing office officials towards oneself (scale of 0-10) | … |
| 35 | satisfaction with patient feedback (scale of 0-10) | … |
| 36 | Satisfaction with availability of medical equipment and facilities (scale of 0-10) | … |
